# Effects of Cyclic Intermittent Hypoxia on ET-1 Responsiveness and Endothelial Dysfunction of Pulmonary Arteries in Rats

**DOI:** 10.1371/journal.pone.0058078

**Published:** 2013-03-05

**Authors:** Zhuo Wang, Ai-Ying Li, Qiu-Hong Guo, Jian-Ping Zhang, Qi An, Ya-jing Guo, Li Chu, J. Woodrow Weiss, En-Sheng Ji

**Affiliations:** 1 Department of Physiology, Hebei Medical University, Shijiazhuang, Hebei, People's Republic of China; 2 Department of Biochemistry, Hebei Medical University, Shijiazhuang, Hebei, People's Republic of China; 3 Department of Pharmacology, Hebei Medical University, Shijiazhuang, Hebei, People's Republic of China; 4 Division of Pulmonary, Critical Care, and Sleep Medicine, Beth Israel Deaconess Medical Center, Boston, Massachusetts, United States of America; The Chinese University of Hong Kong, Hong Kong

## Abstract

Obstructive sleep apnoea (OSA) is a risk factor for cardiovascular disorders and in some cases is complication of pulmonary hypertension. We simulated OSA by exposing rats to cyclic intermittent hypoxia (CIH) to investigate its effect on pulmonary vascular endothelial dysfunction. Sprague-Dawley Rats were exposed to CIH (FiO_2_ 9% for 1 min, repeated every 2 min for 8 h/day, 7 days/wk for 3 wk), and the pulmonary arteries of normoxia and CIH treated rats were analyzed for expression of endothelin-1 (ET-1) and ET receptors by histological, immunohistochemical, RT-PCR and Western Blot analyses, as well as for contractility in response to ET-1. In the pulmonary arteries, ET-1 expression was increased, and ET-1 more potently elicited constriction of the pulmonary artery in CIH rats than in normoxic rats. Exposure to CIH induced marked endothelial cell damage associated with a functional decrease of endothelium-dependent vasodilatation in the pulmonary artery. Compared with normoxic rats, ET_A_ receptor expression was increased in smooth muscle cells of the CIH rats, while the expression of ET_B_ receptors was decreased in endothelial cells. These results demonstrated endothelium-dependent vasodilation was impaired and the vasoconstrictor responsiveness increased by CIH. The increased responsiveness to ET-1 induced by intermittent hypoxia in pulmonary arteries of rats was due to increased expression of ET_A_ receptors predominantly, meanwhile, decreased expression of ET_B_ receptors in the endothelium may also participate in it.

## Introduction

Humans are exposed to hypoxia in a variety of circumstances. Frequently, the exposure is continuous, as with exposure to altitude, but often the exposure is intermittent, particularly with certain disease states. Of the disease states that provide exposure to intermittent hypoxia, the most prevalent is obstructive sleep apnoea (OSA), a condition affecting as many as 10% of working age males and 4% of working age females [1], [2]. When exposed to intermittent hypoxia for several hours per day to mimic OSA, animals have been shown to develop sustained pulmonary hypertension and pulmonary vascular remodeling within a few weeks [3], [4]. Therefore, intermittent hypoxia is a risk factor for cardiovascular disorders and in some cases is a complication of pulmonary vascular diseases [4], [5], as the endothelium is affected [6].

Previous studies have shown that intermittent hypoxia is associated with an elevated plasma endothelin-1 (ET-1) level [7], endothelial dysfunction [8] and augmented vasoconstriction [9], [10]. The endothelium may play an important local regulatory role by producing a number of biologically active substances, including ET-1 and nitric oxide (NO), that participate in the regulation of vascular tone, cell growth, inflammation, and thrombosis [11]. Diminished production of NO and aggravated release of ET-1 are believed to be key initiators of endothelial injury [12].

As a potent endogenous vasoconstrictor, ET-1 is a 21-amino acid peptide that acts via two receptor subtypes, ET_A_ and ET_B_ [13]. Although also made by other cell types, the dominant producers of ET-1 in the vasculature are endothelial cells. ET-1 has been implicated in the pathology of pulmonary arterial hypertension [14]. Activation of the ET-1 system has been demonstrated in both plasma and lung tissues from animal models of pulmonary hypertension, and ET receptor antagonists are effective in improving the condition [15], [16]. Notably, ET-1 immunoreactivity and ET-1 mRNA expression are increased in plasma and lung specimens of patients with pulmonary hypertension [17], [18]. Therefore, elevated levels of ET-1 combined with increased pulmonary vasoconstrictor responses to this peptide may contribute to vascular pathologies in sleep apnea, and it is important to know how vascular pathologies contribute to the augmented constrictor sensitivity.

NO, a potent vasodilatory substance, is generated from _L_-arginine by endothelial nitric oxide synthase (eNOS). Reduced activation of eNOS and reduced generation and bioavailability of NO are characteristic of vascular endothelial dysfunction [19]. It is interesting that ET-1 and NO work as negative feedback signals for each other [20], each one acting to limit the action of the other. It is, therefore, possible that ET-1 contributes to endothelial dysfunction both directly through its vasoconstrictor effects and indirectly through inhibition of NO production.

The aim of the present study was to investigate the effect of cyclic intermittent hypoxia (CIH) on pulmonary arteries in rats. An impaired endothelium-dependent vasodilation and an increased ET-1 responsiveness induced by CIH were observed. These phenomena were further described by changes of vessel tension and expression of ET receptors.

## Methods

### Ethical Approval

All procedures involving animals were conducted in accordance with the National Institute of Health Guide for Care and Use of Laboratory Animals and were approved by the animal Ethics and Use Committee of Hebei Science and Technical Bureau in the People’s Republic of China.

### Animals

All experiments were performed on male Sprague-Dawley rats weighing 180–190 g at entry into the protocol. Rats were housed in normal rat cages with a 12∶12-h light-dark cycle and were given food and water *ad libitum*.

### Drugs

ET-1 and BQ123 were obtained from Alexis Biochemicals (San Diego, CA), and BQ-788 was from Tocris Bioscience (Bristol, UK). Acetylcholine (ACh), phenylephrine (PE) and N^ω^-nitro-L-arginine methyl ester (L-NAME) were purchased from Sigma (St. Louis, MO, USA). ET-1, BQ123, ACh, PE and L-NAME were dissolved in normal saline and stored at −20°C. BQ788 was dissolved in dimethylsulfoxide (DMSO), with the final concentration of DMSO being less than 0.01%. Preliminary experiments showed that <0.01% DMSO did not affect contraction in response to ET-1.

### Cyclic Intermittent Hypoxic Exposure

During hypoxic exposure, animals were placed daily in commercial hypoxic chambers that were ﬂushed with 100% N_2_ to inspired O_2_ fraction (FIO_2_) nadir of 9% for 1 min. The FIO_2_ gradually returned to 21% over the remainder of each cycle. The exposure cycle was repeated every 2 min for 8 h/day, 7 days/wk for 3 wk during the animal’s sleeping hours [21]. Sham control animals underwent identical handling and exposure, but chambers were ﬂushed with room air (normoxia) rather than N_2_. After the exposure cycle was completed, animals were randomly assigned to either physiological investigation or molecular studies.

### Vessel Tension Study

#### Isolation of pulmonary arteries

CIH and sham rats were sacrificed with an overdose of pentobarbital sodium (100 mg/kg im). The heart and lungs were removed and placed in a glass dish containing chilled physiological salt solution [PSS, in mM: 118.3 NaCl, 4.7 KCl, 2.5 CaCl_2_, 1.2 MgSO_4_, 1.2 KH_2_PO_4_, 25.0 NaHCO_3_, and 11.1 glucose]. The second generation pulmonary arteries (1∼2 mm ID) were dissected from the vascular arcade and placed in fresh PSS oxygenated with 95% O_2_–5% CO_2_, where they were cut into rings (2–3 mm long). We mechanically removed endothelium from some vessels by inserting the tips of a watchmaker’s forceps into the lumen of the vessel and rolling the vessel back and forth on saline-saturated filter paper.

#### Contraction and detection of endothelium

Pulmonary Artery rings were suspended between two stainless steel hooks in an organ chamber filled with 7.5 ml of PSS maintained at 37°C and bubbled with 95% O_2_–5% CO_2_. The hooks were attached on one side to a rigid plexiglas support and on the other side to a force transducer (Grass FT03), which was connected to a recording-and-analysis system (Powerlab system, Australia) that fed into the computer.

At the beginning of the experiment, each vessel ring was stretched to its optimal resting tension by stretching, in 0.1-g increments, until the active contraction of the vessel ring to 70 mM KCl reached a plateau. The optimal resting tension of pulmonary arteries was 1.0±0.01 g (n = 30). The rings were then stretched with 1.0 g of passive tension and equilibrated for 90 min. After equilibration, viability was confirmed by contraction to PE (10^−6^ M), and vasorelaxant responses to the endothelial-dependent vasodilator ACh were tested.

#### Concentration-response curves

Tissues were washed repeatedly over 60 min to remove PE and ACh and then exposed to increasing concentrations of ET-1 (0.01 to 100 nM) in a cumulative fashion. Isometric tension was monitored continuously and was expressed as grams per millimeter of vessel length. Thereafter, we chose an appropriate dose to observe the effect of antagonist or inhibitors of vascular responsiveness to ET-1.

#### Effect of antagonists or inhibitors of vascular responsiveness to ET-1

Pulmonary artery rings were pre-contracted with ET-1(3 nM). After the maximal pre-contraction, the vessels were washed with PSS until the line recovered to the basic level. Then, by pre-treatment with BQ123 (a selective ET_A_ receptor antagonist, 10 µM) for 20 min, the rings were contracted with the same concentration of ET-1 again. The effects of BQ788 (a selective ET_B_ receptor antagonist, 10 µM) and L-NAME (an inhibitor of NOS, 10 µM) were observed using the same process.

### Molecular Studies

#### Immunohistochemistry and hematoxylin and eosin (H&E) staining

Pulmonary arteries from the normoxia and hypoxia groups were fixed in 10% (v/v) formalin in 50 mM potassium phosphate buffer (pH 7.0) for 24 h at 4°C. After fixation, the tissues were washed and dehydrated serially in alcohol solutions of ascending concentrations (30%, 50%, 70%, 90% and 100%) and finally cleared in xylene. The tissues were then embedded in molten paraffin wax. Sections were cut at 5 µm thickness, stained with H&E and observed under an optical microscope (Olympus Japan Co., Tokyo, Japan) at a magnification of 400× for histological changes.

The expression and localization of ET-1, ET_A_ receptor and ET_B_ receptor were analyzed by immunohistochemistry. Endogenous peroxidase activity was inhibited by incubation with O.3% hydrogen peroxide. After blocking with 5% (w/v) bovine serum albumin (BSA), the sections were then treated with primary rabbit ET-1, ET_A_ or ET_B_ polyclonal antibodies (Santa Cruz Biotechnology lnc., Santa Cruz, USA.) in phosphate buffered saline (PBS) containing 1% serum (overnight at 4°C), biotin-labeled secondary antibody (30 min at room temperature) and then avidin-biotin-peroxidase complex (Vector Laboratories, Burlingame, CA, USA.). The sections were stained using a diaminobenzidine (DAB) substrate kit (Vector Laboratories, Burlingame, USA.) and the nuclei were counterstained with hematoxylin. The expression and localization of ET-1, ET_A_ and ET_B_ was determined by microscopic observation of the brown peroxidase pulmonary artery.

#### Transmission electron microscopy (TEM)

The pulmonary artery was cut into sections of about 1 mm in length, fixed in 2.5% glutaraldehyde in 0.1 M sodium phosphate buffer, pH 7.4, for 3 h at 4°C and osmicated in 1% osmium tetroxide for 1 h at 4°C. After dehydration with a graded ethanol series, the sample was embedded in Epon 812, and thin sections was cut with a Leica EM UC6 (Leica Co, Vienna, Austria) ultramicrotome. The section was viewed and photographed on a Hitachi 7650 TEM (Hitachi, Japan) at 80 kV.

#### Detection of ET-1, ET_A_, ET_B_ and eNOS expression using western blot

Frozen myocardial tissues were homogenized and lysed with RIPA lysis buffer (Sigma Chemical Co.) containing 100 mg/ml PMSF and 1 mg/ml aprotinin. The lysate was centrifuged, kept on ice for 20 min and centrifuged at 12,000 g at 4°C for 10min to remove the insoluble material. Supernatants were collected to detect ET-1, ET_A_ receptor, ET_B_ receptor, eNOS and GAPDH. Brieﬂy, equal amounts of protein (100–150 µg) were loaded and separated on 8% SDS-PAGE gel, and then transferred to a nitrocellulose membrane (Millipore, USA). After blocking with non-fat milk, the membrane was incubated with primary antibodies ET-1 (Genetex; 1∶500), ET_A_ (Enzo; 1∶1,000), ET_B_ (Enzo; 1∶1,000) or eNOS (BD; 1∶1,000) overnight at 4°C. After washing with PBS-T, the membrane was incubated with goat anti-rabbit secondary antibody (Amersham Biosciences; 1∶5,000) conjugated to horseradish peroxidase for 2 h at room temperature and visualized by an ECL system (Fuji, Japan).

### Measurement of NO in Pulmonary Artery

The concentration of NO was determined on fresh tissue homogenate by measuring the total nitrate and nitrite concentrations (Jian Cheng Biological Engineering Institute, Nanjing, China). This assay determined total NO based on the enzymatic conversion of nitrate to nitrite by nitrate reductase. The reaction was followed by colorimetric detection of nitrite as an azo dye product of the Griess reaction. The absorbance of the mixture at 550 nm was determined with a microplate reader.

### Statistical Analysis

Results were expressed as means±SD, for n specimen of plumonary artery. Data were statistically analyzed using one-way ANOVA followed by a Dunnett post-test. P-values lower than 0.05 were considered significant. Analyses were performed using OriginPro 7.5 (Origin Lab, USA) and EC50 values were determined using the same software from data fitted with a non-linear function.

## Results

### Endothelial Function

**Figure 1:**
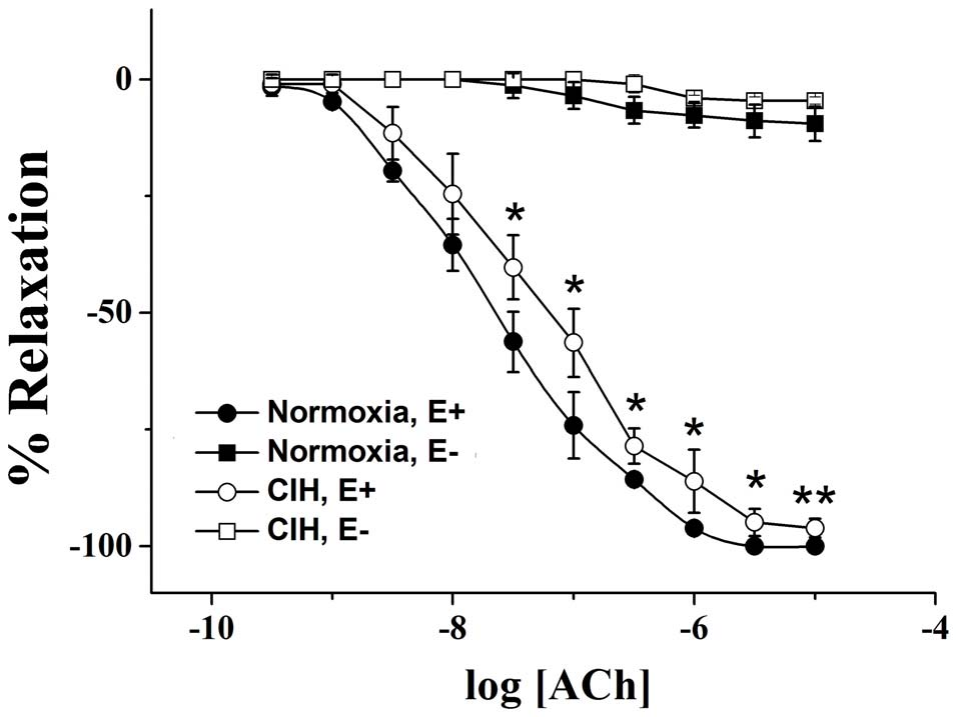
Effects of Ach on relaxation of rings in E+ and E− of normoxic and CIH rats pulmonary arteries. Pulmonary artery rings were pre-contracted with PE (10^−6 ^M), and relaxation to ACh was detected. Relaxant responses to ACh were expressed as a percentage of PE-induced tone. Data were means ± SD; n = 6. ** ***P***<0.01, * ***P***<0.05, relative to normoxia, E+.

In order to determine whether CIH affected endothelium-dependent vasodilatation in pulmonary arteries, the relaxant responses to ACh were examined. The responses to ACh in endothelium-denuded and endothelium-intact pulmonary arteries from normoxic and CIH rats were shown in Figure 1. In pulmonary arteries of every group, ACh relaxed the PE-induced contraction, and the relaxation effects were attenuated in endothelium-denuded rings, no matter from normoxia or CIH rats. In pulmonary arteries from CIH rats, the ACh-induced relaxation was significantly decreased when compared with endothelium-intact normoxia rats, indicating that endothelial function was damaged. Of course, the relaxation was greater than that in the endothelium-denuded pulmonary arteries.

### Contractile Response to ET-1

**Figure 2:**
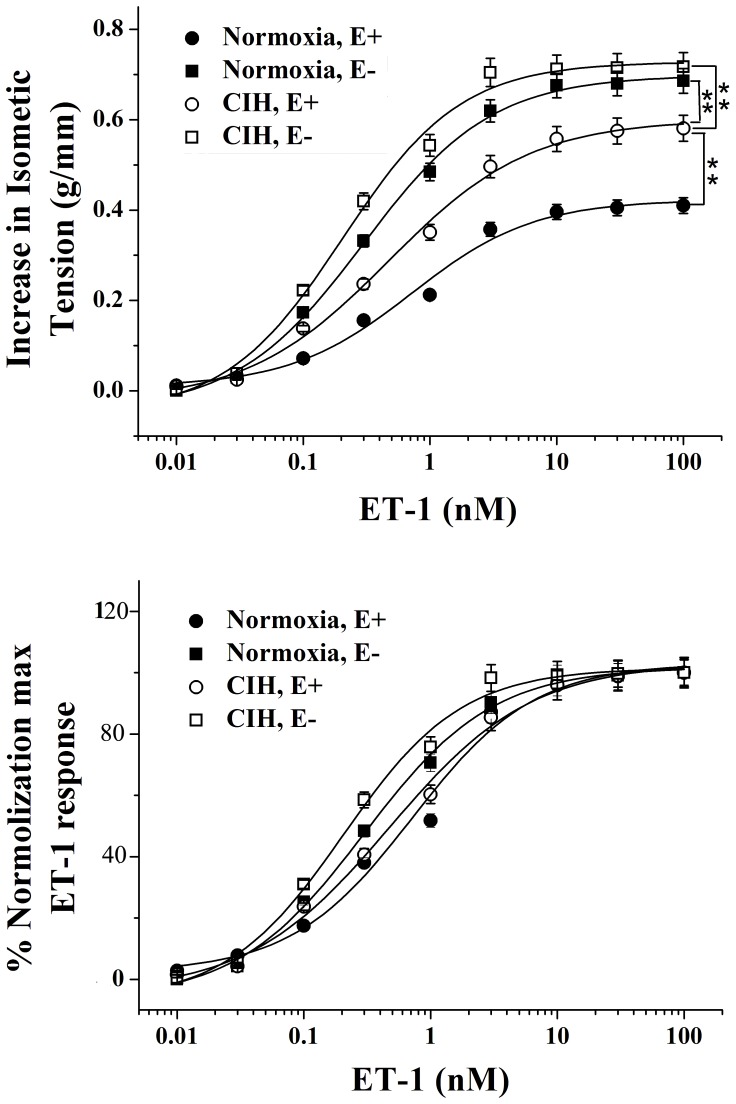
Effects of ET-1 on endothelium-intact (E+) and endothelium-denuded (E−) pulmonary arteries from normoxia and CIH rats. Contraction was expressed as an isometric tension (A) and a normalized isometric tension. The Emax was 0.42±0.02 nmol/L for Normoxia (E+), 0.69±0.01 nmol/L for CIH (E+), 0.60±0.02 nmol/L for Normoxia (E−) and 0.73±0.02 for CIH (E−), respectively. The EC50 was 0.72±0.17 nmol/L for Normoxia (E+), 0.47±0.08 nmol/L for CIH (E+), 0.30±0.03 nmol/L for Normoxia (E−) and 0.21±0.04 for CIH (E−), respectively. ** ***P***<0.01.

ET-1 caused a potent and long lasting contraction of the rat pulmonary artery. The contraction developed slowly, was concentration-dependent and sustained after reaching its maximal amplitude (Fig. 2). As shown in Figure 2A, the contractile efficacy (Emax) of ET-1 underwent an increase (CIH, E−>Normoxia, E−>CIH, E+>Normoxia, E+).The normalized dose-response curves of ET-1 were slightly shifted to the left by both CIH exposure and endothelium removal, or by a combination of the two. The EC50 value of ET-1 of endothelium-intact rings from CIH rats (0.47±0.08 nM, n = 6) was decreased compared with that from normoxia rats (0.72±0.17 nM, n = 6), but increased compared with endothelium-denuded rings from normoxia rats (0.30±0.03 nM, n = 6). The application of CIH exposure and endothelium removal in combination rised the response to ET-1 more efficiently (EC50 = 0.21±0.04 nM, n = 6) (Fig. 2B).

**Figure 3:**
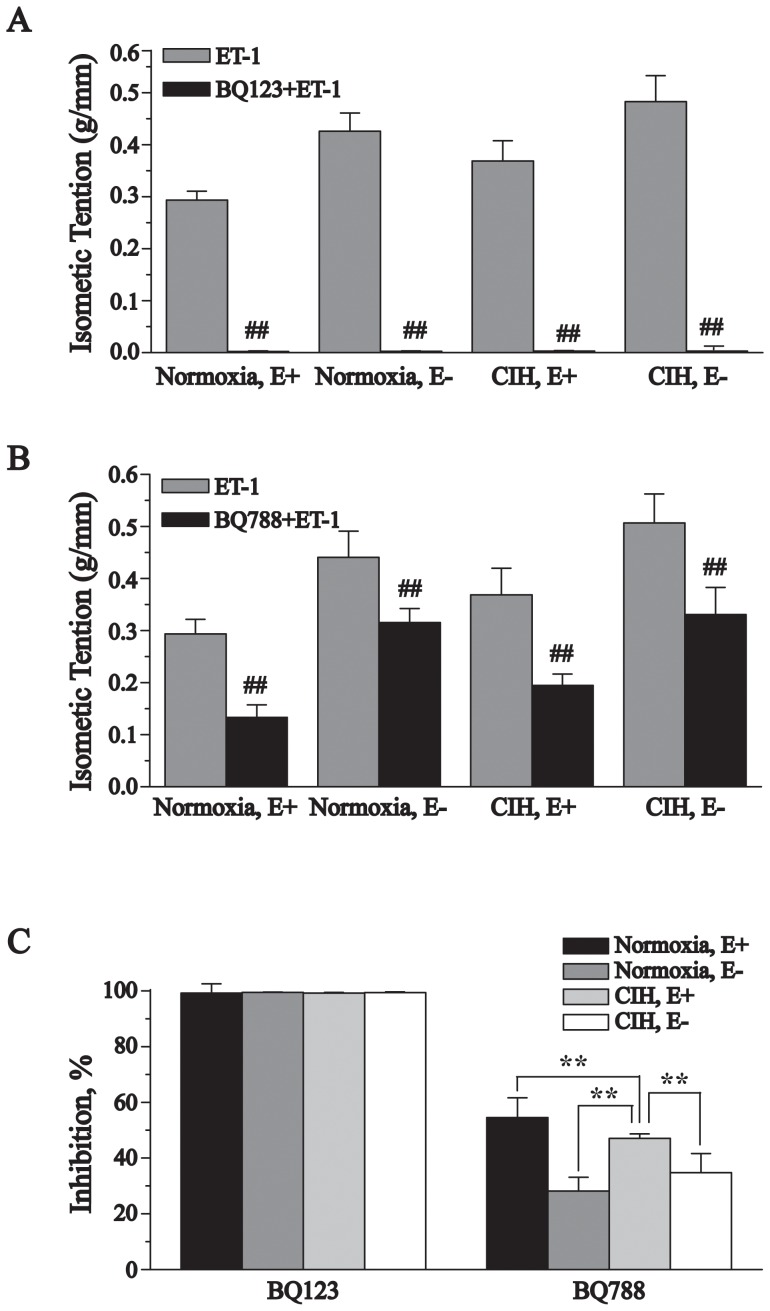
Effects of BQ-123 and BQ-788 on contraction of rat pulmonary arteries to ET-1. Results were expressed as an isometric tension (A and B) and the percentage of the difference to pre-contraction (C). Data were means ± SD; n = 6. **^##^** ***P***<0.01, compared with pre-contraction with ET-1., ** ***P***<0.01.

The ET_A_ receptor antagonist BQ123 nearly completely inhibited ET-1-mediated contractions in pulmonary arteries of endothelium-denuded and endothelium-intact from normoxic rats and CIH rats (Fig. 3A), and the inhibition was not significantly different between the various groups (Fig. 3C). Likewise, the ET_B_ receptor antagonist BQ788 also significantly inhibited ET-1-mediated contractions in four groups of pulmonary arteries (Fig. 3B). Compared with the endothelium-intact normoxic rat pulmonary arteries, this inhibition was markedly decreased in the normoxic endothelium-denuded group and to a lesser extent in the CIH group. Meanwhile, the inhibitory effects of BQ788 in endothelium-denuded rings from CIH rats was larger than that from normoxia rats, but smaller than endothelium-intact rings from CIH rats (Fig. 3C).

**Figure 4:**
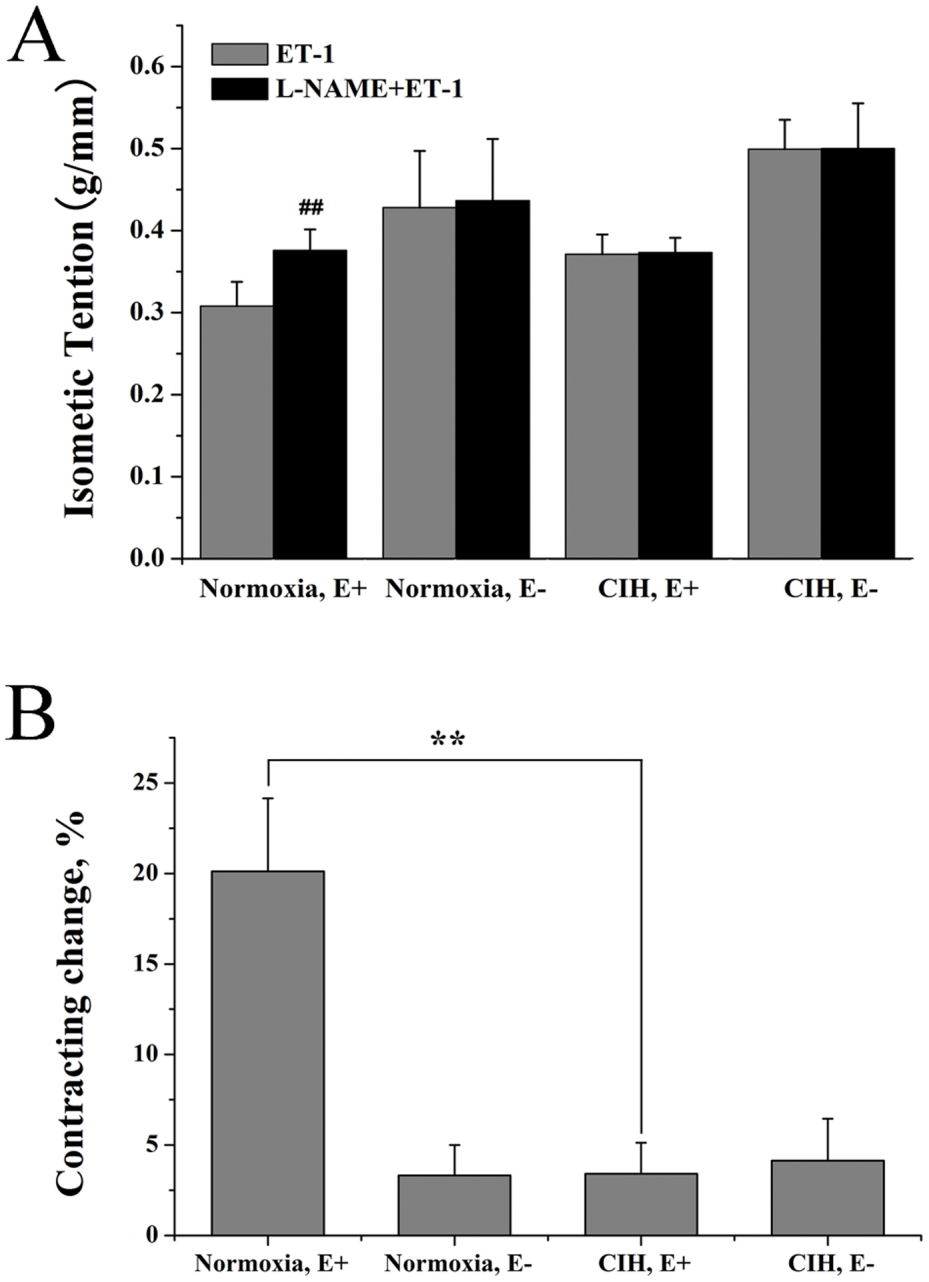
Effects of L-NAME on contraction of rats pulmonary arteries to ET-1. Results were expressed as an isometric tension (A) and the percentage of the difference to pre-contraction (B). Data were means ± SD; n = 6. **^##^** ***P***<0.01, compared with pre-contraction with ET-1., ** ***P***<0.01.

As shown in Figure 4, pre-treatment with L-NAME significantly augmented the contraction caused by ET-1 in the normoxic endothelium-intact rings. However, after removal of the endothelium, or CIH exposure, the contraction induced by ET-1 was not affected by L-NAME.

### Morphological and Histological Changes in Endothelial Tissues of CIH Rats

**Figure 5:**
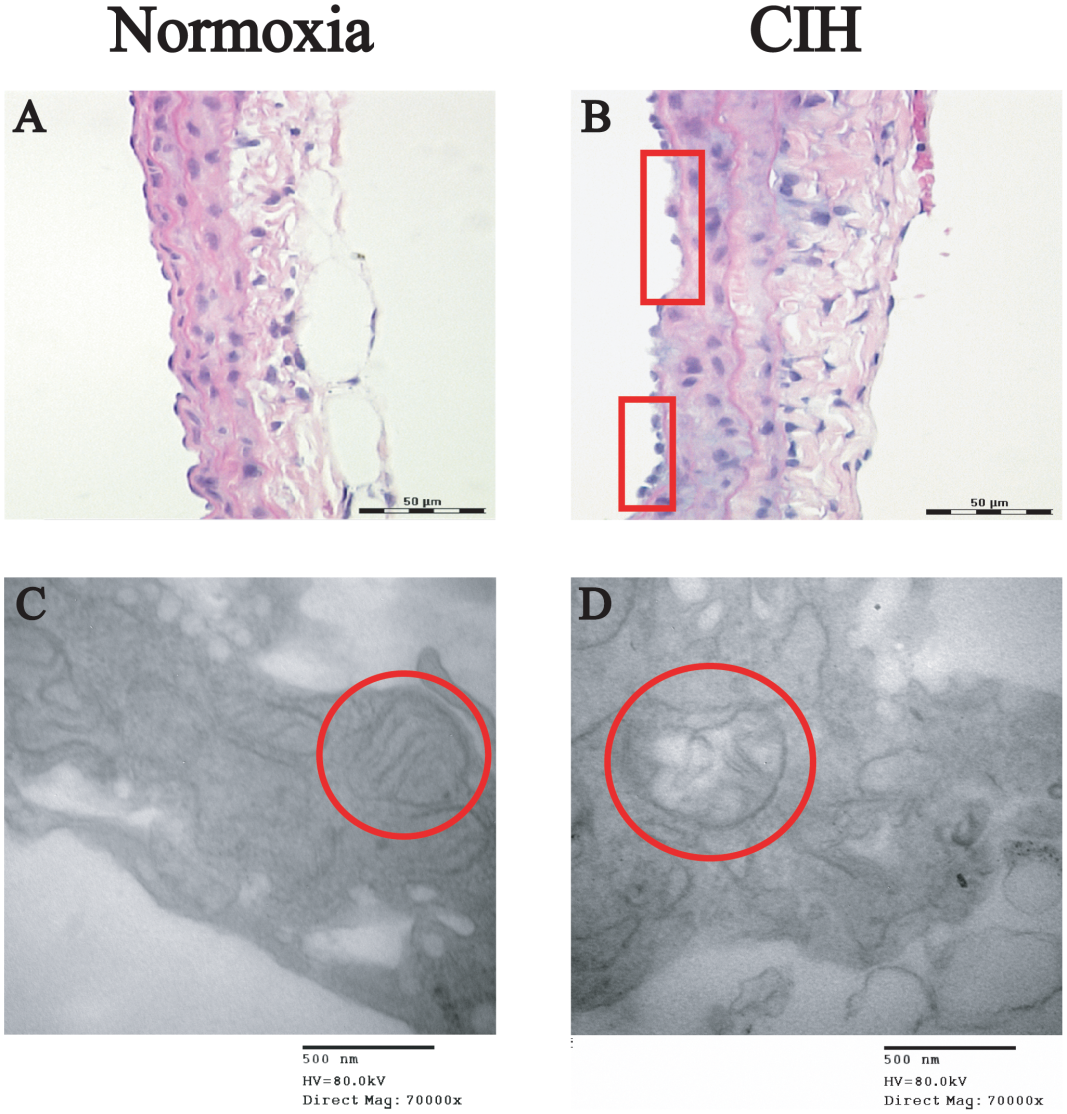
Effects of CIH on pulmonary arteries histopathological changes. Representative microscopic photographs of pulmonary arteries stained with H&E (magnification 400×) (A and B) and TEM (C and D). Pulmonary arteries obtained from normoxia group (A and C) and CIH group (B and D). In the pulmonary artery segments from the CIH group, there were histopathological changes of the endothelial monolayer with cellular enlargement and edema, denudation of some endothelial cells. The ultrastructure was observed by TEM in the (C) normoxia group and (D) CIH group. Large defects of the endothelial layer, cellular vacuolation and mitochondrial damage could be observed in D.

Endothelia of rat pulmonary arteries were histopathologically examined by light and electron microscopy. A normal tissue architecture of the endothelium was seen in the normoxia group, comprised of a complete endothelial monolayer, regularly shaped and oriented endothelial cells and normal mitochondria (Fig. 5A and C). Rat pulmonary arteries in the CIH group exhibited mild histopathological changes of the endothelial monolayer with cellular enlargement and edema, and denudation of some endothelial cells (Fig. 5B). Large defects of the endothelial layer, cellular vacuolation and mitochondrial damage could be observed by TEM (Fig. 5D).

### Effects of CIH on Pulmonary Artery Expression and Localization of ET-1, ET_A_ and ET_B_

**Figure 6:**
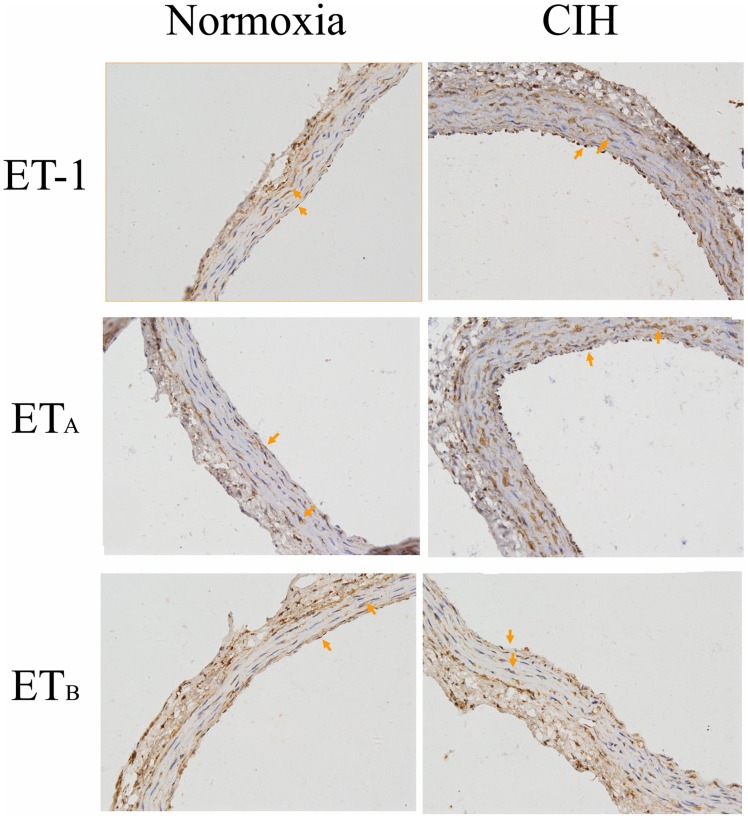
Effects of CIH on ET-1, ETA receptor and ETB receptor localization in pulmonary arteries were analyzed by immunohistochemistry. Immunoreactivity for ET-1 and ETA receptor increased in the media and intima of the pulmonary artery after exposure to CIH. Meanwhile, immunoreactivity for the ET_B_ receptor decreased markedly in the intima and there was no significant change in the media of the pulmonary artery after exposure to CIH.

**Figure 7:**
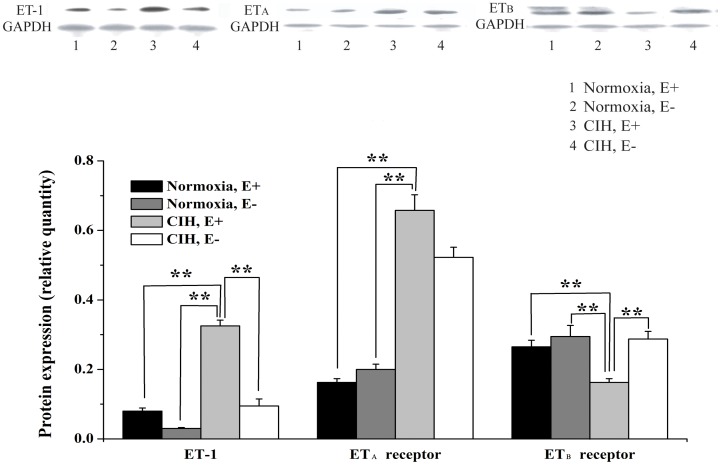
Effects of CIH on ET-1, ET_A_ receptor and ET_B_ receptor expression in pulmonary arteries. Equal loading of protein was confirmed using an anti-GAPDH antibody. Quantification of the protein expression was normalized to GAPDH. ** ***P***<0.01.

By immunohistochemical analysis, ET-1 and the ET-1 receptors were detected in the media and intima of pulmonary arteries (Fig. 6). In normal rats, ET-1 was predominantly distributed in the media of the pulmonary artery, and the immunoreactivity of ET-1 increased after exposure to CIH. The ET_A_ receptor was predominantly distributed in the media with faint staining in the intima of the pulmonary artery in normal rats. Meanwhile, exposure to CIH increased the immunoreactivity for the ET_A_ receptor in the media of the pulmonary artery. Positive staining for ET_B_ receptors was also predominantly observed in the intima with weak staining in the media of the normal rat pulmonary artery. Unlike with the ET_A_ receptor, immunoreactivity for ET_B_ receptor markedly decreased in the intima and no significant change in the media of the pulmonary artery after exposure to CIH.In the pulmonary arteries from the CIH, E+ group, a dramatic increase of ET-1 and ET_A_ receptor compared with the normoxia, E+ group was detected (*P*<0.01). Removal of the endothelium decreased the expression of ET-1 (*P*<0.01), but did not affect the expression of ET_A_ receptor. Exposure to CIH significantly suppressed ET_B_ receptor protein level in endothelium-intact, but not endothelium-denuded, rat pulmonary arteries (Fig. 7).

### CIH Reduces Rat Pulmonary NO Production and eNOS Expression

**Figure 8:**
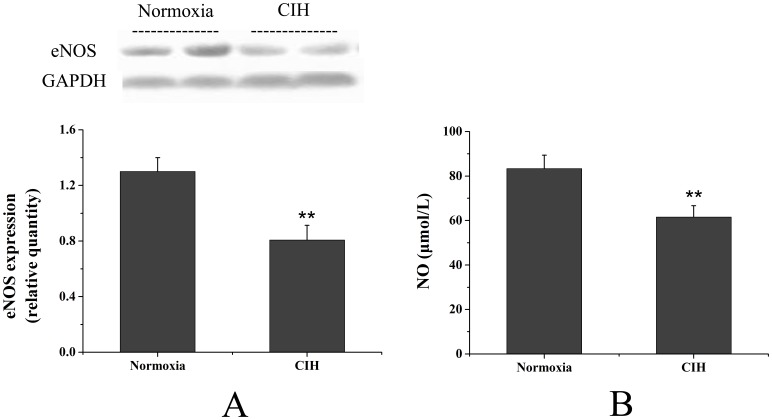
Effects of CIH on eNOS expression and NO level in pulmonary arteries. Effects of CIH on eNOS expression in pulmonary arteries were analyzed by western blot (A). Equal loading of protein was confirmed using an anti-GAPDH antibody. Quantification of the eNOS expression was normalized to GAPDH. The level of NO in the pulmonary artery (B) in CIH rats was dramatically reduced, compared with the normoxia group. Results are presented as means ± SD (n = 6). ** ***P***<0.01, compared with normoxia group.

As shown in Figure 8, the concentration of NO in the pulmonary artery of CIH rats was dramatically reduced, compared with the normoxia group (*P*<0.01). By western blot analysis, the expression of eNOS in the pulmonary artery of CIH rats (normalized to GAPDH) was also dramatically reduced, compared with that of the normoxia group (*P*<0.01).

## Discussion

The results of our investigation have shown that intermittent hypoxia caused marked changes in functional and morphological properties of pulmonary arteries in rats, especially in the endothelium. Exposure to intermittent hypoxia was shown to decrease the relaxation to ACh in pulmonary arteries pre-treated with PE. In addition, ET-1 was found to induce a significant dose-dependent contraction of the pulmonary artery, and vessels from CIH rats were more sensitive to ET-1 than those from normoxia rats. Thus, the hypoxic conditions used (2 min. cycles of 9%/21% O_2_, 8 h/day, 3 wks) eventually impaired endothelium-dependent vasodilation and increased vasoconstrictor responsiveness, which is in agreement with the pathology observed in human OSA [22], [23]. Moreover, the response to ET-1 in the pulmonary artery of CIH rats was higher than in endothelium-intact artery rings and lower than that of endothelium-denuded artery rings. Thus, it appears that the increased vasoconstrictor responsiveness induced by intermittent hypoxia was endothelium dependent. The endothelium releases diverse vasoactive mediators, including ET-1 and NO, which regulate the physical and biochemical properties of the pulmonary vessels and affect vascular contractility. In healthy individuals, a balance between these mediators is thought to mediate the low basal pulmonary vascular tone [24]. For this reason we have examined, and shown, that intermittent hypoxia decreased NO production and eNOS protein expression, while increasing ET-1 expression in the rat pulmonary artery. Such an imbalance pre-disposes the vasculature to increased tone, altered remodeling, proliferation and endothelial injury [12], [25].

As reported previously, ET-1, a potent pulmonary vasoconstrictor, acts via two receptor subtypes, ET_A_ and ET_B_ [26]. It is believed that the ET_A_ receptor is mainly located on vascular smooth muscle cells, and it is the principal subtype for ET-1-induced pulmonary vasoconstriction [27], [28], [29]. ET_B_ receptors are expressed predominantly in endothelial cells and at lower levels in vascular smooth muscle cells [6]. Stimulation of the endothelial ET_B_ receptor results in release of NO and prostacyclin [30] causing vasodilatation, whereas stimulation of the ET_B_ receptor in the vascular smooth muscle cell results in vasoconstriction. Thus, the net effect produced by ET-1 is determined by the balance between ET_A_ and ET_B_ receptors and their localization. Under physiological conditions, the net effect is vasoconstriction mediated by the ET_A_ receptor, which is partly counteracted by ET_B_ receptor-mediated release of NO and prostacyclin. However, under certain pathophysiological conditions, the response to ET receptor antagonists may be changed [14].

Our results are supportive of the principal vasoconstrictor role for ET_A_ receptors and a vasodilator role for endothelial ET_B_ receptors in ET-1-induced pulmonary vasoconstriction. Additionally, the elevated responsiveness of ET-1 in pulmonary arteries of rats induced by intermittent hypoxia may be due to both increased ET_A_ receptor expression and decreased expression of endothelial ET_B_ receptor. This conclusion is based on several lines of evidence. 1) The contraction induced by ET-1 was nearly completely abolished by pre-treatment with BQ123, and this effect was not influenced by removal of the endothelium or exposure to CIH. 2) BQ-788 only partially inhibited the pulmonary artery contraction caused by ET-1. The inhibition was markedly decreased both in the normoxic endothelium-denuded and CIH group, while the decrease of contraction in the endothelium-denuded rings was larger than that of the CIH group. These results indicated that the effect of intermittent hypoxia on the ET_B_ receptor may be associated with endothelium dysfunction. 3) Analysis of expression and localization of ET receptors by western blot and immunohistochemistry showed that intermittent hypoxia induced an increase of ET_A_ receptors mainly in the media of the pulmonary artery, while it decreased expression and immunoreactive protein concentration of ET_B_ receptors in the intima of the pulmonary artery, and no significant change in media. These findings suggest that increased ET_A_ receptor levels play an important role in the pathogenesis of CIH, which mediates the augmented sensitivity to ET-1. Meanwhile, decreased expression of the endothelial ET_B_ receptor may indirectly augment the response to ET-1 through reducing production of NO in the pathogenesis of CIH.

In addition, blockade of ET_B_ receptors by BQ-788 has been associated with an increase in the plasma concentration of ET-1 [31]. Similarly, ET_B_ receptor–deficient transgenic rats were found to have an increased plasma ET-1 concentration along with higher basal pulmonary arterial pressure compared with control rats [32]. Furthermore, Pollock et al. [33] reported that although ET_B_ receptor-deficient rats have similar basal mean arterial pressure to the wild-type rats, they exhibit enhanced pressor responses to ET-1 compared with control rats. These findings suggest that ET_B_ receptors may also function as clearance receptors for ET-1, and in their absence allows the ET-1 concentration to increase, thereby causing greater constriction through the activation of ET_A_ receptors. Accordingly, the decreased expression of ET_B_ receptors observed in our study may also account for the increased ET-1 protein level and augmentation of ET-1-induced vasoconstriction of pulmonary arteries after exposure to intermittent hypoxia in rats.

Consistent with the report that NO production attenuates the vasoconstrictor response to ET-1 [34], [35], [36], [37], our study showed that L-NAME could significantly augment the contraction caused by ET-1 in the pulmonary artery. Because ET-1 may mediate a concomitant endothelial NO release [38], [39], [40], the contractile response was evaluated in the presence of L-NAME, which has been previously shown to abolish the endothelium-dependent relaxation induced by ACh in major mouse blood vessels [41], [42]. However, the impaired NO-dependent vasodilatation induced by CIH may change the response of L-NAME to ET-1 in the pulmonary artery. These results indicated that the increased sensitivity to ET-1 induced by CIH was at least partly independent of NO production. However, the complex interactions between ET-1 and NO pathways may also contribute to these results. Endothelial-derived NO has been described to reduce ET-1 production in the porcine aorta *in vitro* [43]. NO inhibition could thereby result in an up-regulation of the ET-1 pathway during CIH.

Overall, this study has established that CIH exposure impaired the endothelium in functional and morphological properties of pulmonary arteries in rats. It also demonstrated that the increased responsiveness to ET-1 of pulmonary artery in rats, which was induced by CIH, was due to increased expression of ET_A_ receptors predominantly, meanwhile, decreased expression of ET_B_ receptors in the endothelium may also participate in it.
